# Complete Genome Sequence of the Hypervirulent Bacterium Clostridium difficile Strain G46, Ribotype 027

**DOI:** 10.1128/genomeA.00073-15

**Published:** 2015-03-26

**Authors:** Tom Gaulton, Raju Misra, Graham Rose, Primo Baybayan, Richard Hall, Jane Freeman, Jane Turton, Steve Picton, Jonas Korlach, Saheer Gharbia, Haroun Shah

**Affiliations:** a Public Health England, London, United Kingdom; b Pacific Biosciences, Menlo Park, California, USA; c Leeds Institute of Molecular Medicine, University of Leeds, Leeds, West Yorkshire, United Kingdom

## Abstract

Clostridium difficile is one of the leading causes of antibiotic-associated diarrhea in health care facilities worldwide. Here, we report the genome sequence of C. difficile strain G46, ribotype 027, isolated from an outbreak in Glamorgan, Wales, in 2006.

## GENOME ANNOUNCEMENT

Clostridium difficile has emerged in recent years as a common cause of infectious antibiotic-associated diarrhea within health care facilities worldwide. It is a Gram-positive anaerobic prokaryote, notorious for being multidrug-resistant and causing C. difficile infections (CDIs). It can produce a variety of virulence factors that lead to intestinal colonization and disease; for example, it is known to express up to three toxins, toxin A (TcdA), toxin B (TcdB), and, less commonly, binary toxin (CDT) (1), which contribute to the clinical spectrum of the disease, ranging from mild nonspecific diarrhea to severe pseudomembranous colitis (PMC) and paralytic ileus (2, 3).

C. difficile has been linked to antibiotic use for decades. During the 1970s, increases in CDIs were attributed to clindamycin use, in the 1980s, to cephalosporin and penicillin use, and in the 2000s, to fluoroquinolone use (4). Careful antimicrobial stewardship has been used to limit CDI risk, but it remains a significant burden on health care systems (5). Therapeutic options for CDI treatment remain limited to metronidazole and vancomycin, with the recent addition of fidaxomicin (6). Worryingly, despite treatment, approximately 20 to 30% of patients suffer from recurrence (7).

The genomes for several 027 ribotyped strains have been sequenced; however, very few have been sequenced and assembled to completion. We report here the complete genome of C. difficile strain G46. This strain was isolated from an outbreak in Glamorgan, Wales, in 2006 and is representative of the “hypervirulent” ribotype 027 strains.

Sequencing was performed by Pacific Biosciences (Menlo Park, CA) using the PacBio RS II system. A total of 146,976 filtered subreads were used for the assembly, from three single-molecule real-time (SMRT) cells. The read lengths varied from 50 to 63,997 bases in length and were assembled using the HGAP long-read assembler. A single circularized chromosomal contig was generated, 4,189,317 bases in length (29% G+C content), and was annotated using the Prokka annotation pipeline (8). We annotated 3,811 protein coding sequences, which encompassed 84.1% of the genome. Of the 3,811 annotated sequences, 706 encoded hypothetical or putative proteins. We identified 125 tRNA-coding, 5 rRNA-coding operons, and 176 signal peptides. In parallel, the epigenome was elucidated, revealing many modifications borne from DNA methylation (9).

Comparisons with the well-characterized C. difficile strain R20291, ribotype 027, revealed high sequence similarity (99.99%). C. difficile G46 possessed both the PaLoc operon (19.6 kb) and the binary toxin operon (6.2 kb), sharing 100% sequence similarity with strain R20291. Like strain R20291, strain G46 encodes for variety of antibiotic resistance features, such as point mutations in the DNA gyrase genes, which cause resistance to fluoroquinolones (10). The MICs of moxifloxacin (fluoroquinolone) corroborated the genomic prediction, demonstrating high resistance, with an MIC of 16 mg/liter (an MIC of 4 mg/liter indicates resistance).

### Nucleotide sequence accession numbers.

The genome sequence of C. difficile strain G46 has been deposited in the ENA under the accession no. CDND00000000. The version described in this paper is the first version, CDND01000000. The methylation motifs were deposited in REBASE (http://rebase.neb.com/rebase/refs/18866.html).
